# Seven-Year PSA ≤ 0.2 ng/mL After High-Dose-Rate Brachytherapy Indicates Eligibility for Discontinuing PSA Surveillance in Prostate Cancer

**DOI:** 10.3390/cancers17193151

**Published:** 2025-09-28

**Authors:** Tomoyuki Makino, Takayuki Sakurai, Shigeyuki Takamatsu, Ryunosuke Nakagawa, Taiki Kamijima, Hiroshi Kano, Renato Naito, Hiroaki Iwamoto, Hiroshi Yaegashi, Kazuyoshi Shigehara, Takahiro Nohara, Kouji Izumi, Atsushi Mizokami

**Affiliations:** 1 Department of Integrative Cancer Therapy and Urology, Kanazawa University Graduate School of Medical Science, 13-1 Takara-Machi, Kanazawa 920-8641, Japan; r_a_rhero0226southern@yahoo.co.jp (R.N.); kamiji0029@yahoo.co.jp (T.K.); kanazawa_iimati@yahoo.co.jp (H.K.); thealfuu@yahoo.co.jp (R.N.); hiroaki017@yahoo.co.jp (H.I.); hyae2002jp@yahoo.co.jp (H.Y.); kshigehara0415@yahoo.co.jp (K.S.); t_nohara704@yahoo.co.jp (T.N.); azuizu2003@yahoo.co.jp (K.I.); mizokami@staff.kanazawa-u.ac.jp (A.M.); 2 Department of Radiology, Kanazawa University Graduate School of Medical Science, 13-1 Takara-Machi, Kanazawa 920-8641, Japan; tkyk81@gmail.com (T.S.); shigerad@staff.kanazawa-u.ac.jp (S.T.)

**Keywords:** high-dose-rate brachytherapy, prostate cancer, PSA monitoring, recurrence

## Abstract

Background: We evaluated the long-term treatment outcomes of patients with clinically localized and locally advanced prostate cancer (PC) who underwent high-dose-rate brachytherapy (HDR-BT) combined with external beam radiotherapy (EBRT). The primary objective was to identify the optimal timing for discontinuing prostate-specific antigen (PSA) monitoring after HDR-BT. Methods: This analysis included 338 patients with PC who received HDR-BT combined with EBRT between 2006 and 2022 and had a minimum follow-up of 5 years. The patients were stratified based on their PSA levels, and factors associated with recurrence were identified. Results: The median observation period was 8.9 years (range, 5.0–19.0 years). The 10-year recurrence-free survival rate was 92.0%, with 26 recurrences. PSA levels at 5 and 7 years were significantly correlated with oncological outcomes after HDR-BT. Multivariate analysis revealed that a PSA level of >0.2 ng/mL at 5 years was an independent poor prognostic factor for recurrence (hazard ratio, 117.57; 95% confidence interval, 6.22–2223.37; *p* = 0.001). No patient with a PSA level of ≤0.2 ng/mL at 7 years developed recurrences. Conclusions: Based on our long-term data, we propose that PSA monitoring may be safely discontinued in patients with a PSA level of ≤0.2 ng/mL 7 years after HDR-BT because the risk of recurrence beyond this point is exceedingly low.

## Simple Summary

Although both domestic and international guidelines provide recommendations for follow-up methods after definitive treatment of prostate cancer, they do not clearly specify when follow-up can be safely discontinued. This retrospective study aimed to determine the optimal timing for cessation of monitoring following high-dose-rate brachytherapy. Findings showed that prostate-specific antigen (PSA) levels at 5 and 7 years post-treatment were significantly associated with oncological outcomes. Notably, no recurrences were observed among patients whose PSA levels remained ≤0.2 ng/mL at 7 years post-treatment. These findings suggest that PSA surveillance may be safely discontinued at 7 years in this population, providing a clear, evidence-based benchmark for ending long-term monitoring. This could reduce patient burden and healthcare costs without compromising safety.

## 1. Introduction

Prostate cancer (PC) remains one of the most commonly diagnosed malignancies among men worldwide, and its incidence continues to rise with the aging population and the widespread implementation of prostate-specific antigen (PSA) screening. For patients with localized disease, multiple definitive treatment options are available, including radical prostatectomy, external beam radiotherapy (EBRT), and brachytherapy (BT). The National Comprehensive Cancer Network (NCCN) guidelines currently recommend BT, either as monotherapy or in combination with EBRT, as an optimal treatment for selected patients with unfavourable intermediate/high-risk and localized/locally advanced PC [1]. Among BT techniques, high-dose-rate BT (HDR-BT) has gained attention owing to its ability to deliver a highly conformal, intense radiation dose to the prostate while minimizing exposure to surrounding normal tissues. This approach enables excellent long-term biochemical control with a favorable toxicity profile, as demonstrated in several clinical studies [2,3,4,5,6]. Long-term oncological outcomes of HDR-BT have been reported, with follow-up periods exceeding 10 years in some studies, confirming durable biochemical control and acceptable safety [7,8,9,10]. However, despite the accumulation of these data, the optimal strategy for post-treatment surveillance in clinical practice remains undefined. While PSA monitoring is a cornerstone of follow-up after definitive therapy, including radiotherapy (RT), there is currently no consensus regarding the appropriate duration or timing for discontinuing PSA assessments in patients with long-term remission. According to the most recent NCCN guidelines [1], PSA measurement is recommended every 6–12 months for the first 5 years and annually thereafter. Nevertheless, the clinical significance of specific PSA time points after HDR-BT, and their utility in predicting long-term recurrence risk or guiding the discontinuation of surveillance, have not been fully elucidated.

Given these gaps in current knowledge, a better understanding of the prognostic value of PSA levels at various post-treatment intervals is warranted. In particular, identifying clinically meaningful time points that indicate minimal recurrence risk could help individualize follow-up schedules, reduce the burden of unnecessary testing, and optimize long-term survivorship care. Therefore, this study aimed to evaluate the clinical utility of PSA measurements at specific time points after HDR-BT by assessing their prognostic significance in the long-term follow-up of patients with PC.

## 2. Materials and Methods

### 2.1. Patient Demographics

Between January 2006 and December 2022, 574 consecutive patients with PC underwent HDR-BT with EBRT at Kanazawa University Hospital. Of the 574 patients, 31, 188, 7, and 10 patients were excluded due to inadequate radiation dose protocols, a follow-up period of <5 years, distant metastases, and no neoadjuvant hormonal therapy (HT), respectively. Thus, we retrospectively analyzed the remaining 338 patients using their medical records. Clinical staging was categorized based on the tumor–node–metastasis (TNM) classification [11].

### 2.2. RT Protocol

**Figure 1:**
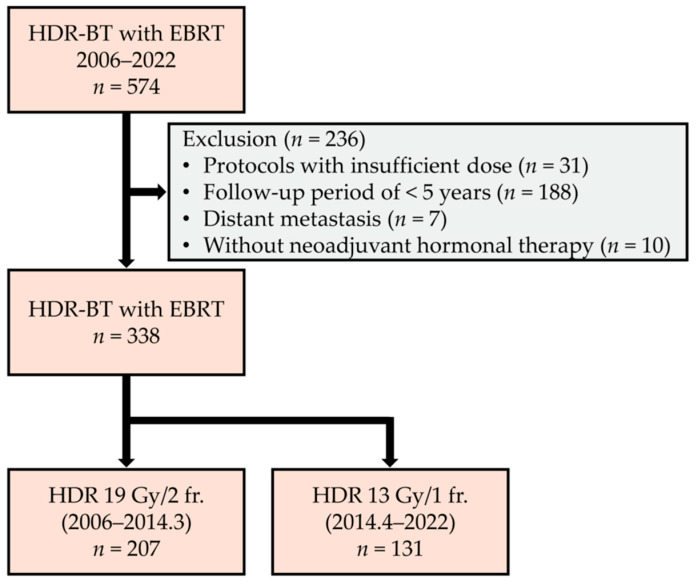
Flow diagram of the patient selection process.

The HDR-BT protocol has been previously reported [12]. In our protocol, before HDR-BT, neoadjuvant HT, consisting of bicalutamide (80 mg/day) plus androgen deprivation therapy (ADT), was administered for 6 months. The ADT regimen included a gonadotropin-releasing hormone agonist—either leuprorelin acetate (11.25 mg) or goserelin acetate (10.8 mg). We recommend adjuvant HT for 2 years for patients with more than two high-risk factors based on localized high-risk categories according to the NCCN (≥cT3 or ≥Grade Group 4 or PSA > 20 ng/mL) or those with locally advanced PC (e.g., ≥cT3b). HDR-BT is recommended for regional lymph node metastasis, regardless of the size or number of nodes; however, HDR-BT is not suitable for metastases outside the pelvic external irradiation field. Intensity-modulated RT was administered to initially deliver EBRT at 46 Gy in 23 fractions, usually 1 or 2 weeks after the BT procedure. The details of our irradiation protocol have been previously documented [12]. All patients were treated with a combined regimen of HDR-BT and EBRT at various fractionations. HDR was administered at a dose of 19 Gy in two fractions (from 2006 to March 2014) and 13 Gy in a single fraction (from April 2014), respectively (Figure 1). In the current protocol, the biologically effective doses were 241 Gy (19 Gy in two fractions) and 233 Gy (13 Gy in a single fraction) using an α/β ratio of 1.5 Gy.

### 2.3. Statistical Analyses

The recurrence-free survival (RFS), cancer-specific survival (CSS), and overall survival (OS) from the first day of BT were calculated using the Kaplan–Meier method and log-rank test. Recurrence was defined as radiographic or biochemical recurrence based on the Phoenix criteria [13] or salvage HT initiation. The time to recurrence was analyzed using the Cox proportional hazards model. Statistical analyses were performed using GraphPad Prism (version 6.07; GraphPad Software Inc., San Diego, CA, USA) and Statistical Package for the Social Sciences (version 29; IBM Corp., Armonk, NY, USA). Two-sided *p*-values < 0.05 were used to denote statistical significance for all analyses.

## 3. Results

### 3.1. Patient Characteristics

This retrospective analysis included 338 patients with PC clinical stages of T1–T4, N0–1, and M0. Table 1 presents the clinical and pathological features of all patients, including those without recurrence at 5 and 7 years after HDR-BT. The overall median follow-up duration was 8.9 years (range, 5.0–19.0 years). Overall, 291 and 195 patients were followed up without recurrence for >5 and 7 years, respectively.

### 3.2. Treatment Outcomes

**Figure 2:**
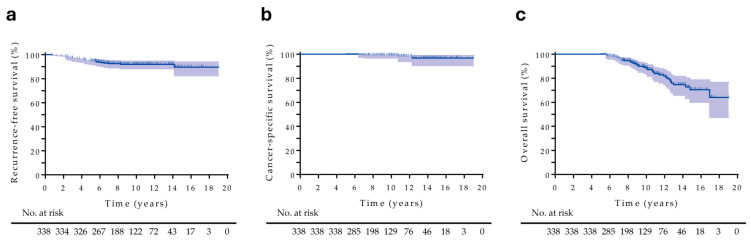
Kaplan–Meier estimate of all patients. (**a**) Recurrence-free survival, (**b**) cancer-specific survival, and (**c**) overall survival.

During the observation period, 26 (7.7%) patients had disease recurrences (4 patients with radiographic recurrences, 18 patients with biochemical recurrences, and 4 patients who experienced initiations of salvage HT). The most frequent recurrence site of radiographic progression was the bone, followed by the lung. Moreover, 42 (12.4%) patients died, including four patients who died from PC. The 5-year RFS, CSS, and OS rates were 94.9% (95% confidence interval [CI], 92.0–96.8), 100%, and 100%, respectively (Figure 2a–c). The corresponding values at 10 years were 92.0% (95% CI, 88.3–94.5), 99.2% (95% CI, 96.8–99.8), and 88.7% (95% CI, 83.6–92.3), respectively. Based on clinical and oncological parameters, the Kaplan–Meier curves indicated significantly lower RFS in patients with higher Grade Group, clinical T stage, clinical N stage, and pre-HDR-BT PSA and inferior RFS with different HDR-BT protocols (Supplementary Figure S1). Univariate and multivariate Cox proportional hazards regression analyses indicated that no factor significantly predicted recurrence in all patients. The only prognostic factor for OS was age (Supplementary Table S1).

### 3.3. Prognostic Factors Associated with RT Recurrences over 5 Years

**Figure 3:**
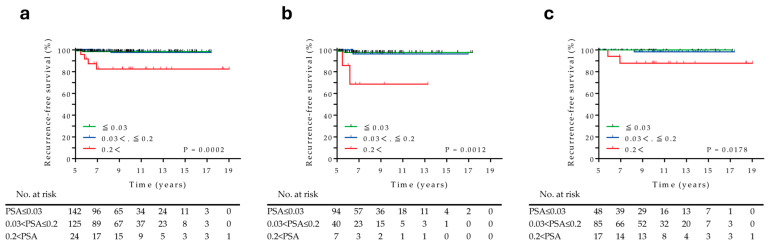
Kaplan–Meier estimate of recurrence-free survival > 5 years after HDR-BT stratified based on the PSA level at 5 years. (**a**) Overall patients, (**b**) patients with and (**c**) without adjuvant hormonal therapy.

In total, 291 patients without recurrence at the 5-year time point were divided into three groups based on their PSA levels: ≤0.03 ng/mL (*n* = 142), 0.03–0.2 ng/mL (*n* = 125), and >0.2 ng/mL (*n* = 24). The overall median follow-up duration was 9.1 years (range, 5.0–19.0 years). During the observation period, eight (2.7%) patients experienced disease recurrences (3 patients with radiographic recurrences, 4 patients with biochemical recurrences, and 1 patient who experienced initiation of salvage HT). The Kaplan–Meier analysis revealed that the overall RFS was significantly poorer in the high PSA group at 5 years (log-rank; *p* = 0.0002) (Figure 3a), and a similar trend was observed irrespective of the presence or absence of adjuvant HT (*p* = 0.0012 and *p* = 0.0178, respectively) (Figure 3b,c). The recurrence rates were 1.4% (*n* = 2), 1.6% (*n* = 2), and 16.7% (*n* = 4) for patients with PSA levels of ≤0.03 ng/mL, >0.03–0.2, and >0.2 ng/mL, respectively (Supplementary Table S2). Univariate and multivariate Cox proportional hazards regression analyses revealed that a PSA level of >0.2 ng/mL was the only significant predictor of recurrence > 5 years after HDR-BT (hazard ratio [HR], 117.57; 95% CI, 6.22–2223.37; *p* = 0.001) (Table 2).

### 3.4. Prognostic Factors Associated with RT Recurrences over 7 Years

**Figure 4:**
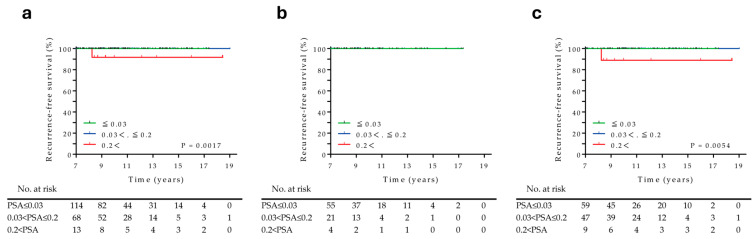
Kaplan–Meier estimate of recurrence-free survival > 7 years after HDR-BT stratified based on the PSA level at 7 years. (**a**) Overall patients, (**b**) patients with and (**c**) without adjuvant hormonal therapy.

In total, 195 patients without recurrence at the 7-year time point were divided into three groups based on their PSA levels: ≤0.03 ng/mL (*n* = 114), 0.03–0.2 ng/mL (*n* = 68), and >0.2 ng/mL (*n* = 13). The overall median follow-up duration was 10.1 years (range, 7.0–19.0 years). During the observation period, only one patient with a PSA level of >0.2 ng/mL experienced recurrence, and this occurred in the group without adjuvant HT (Figure 4a,c). Interestingly, patients without recurrence 7 years after HDR-BT did not experience any subsequent recurrence in the adjuvant HT group, regardless of their PSA levels (Figure 4b). The proportion of patients who ultimately developed recurrence was 0%, 0%, and 7.7% for those with PSA levels of ≤0.03 ng/mL, 0.03–0.2, and >0.2 ng/mL, respectively (Supplementary Table S3). Therefore, a PSA level of ≤0.2 ng/mL 7 years after HDR-BT indicated no subsequent recurrence.

## 4. Discussion

Post-treatment surveillance for PC is a critical component of long-term patient management, yet the optimal strategy remains controversial. According to the National Cancer Center of Japan, routine follow-up visits are recommended every 3 months for the first 2 years, every 6 months for the subsequent 2 years, and annually thereafter [14]. In contrast, the latest NCCN guidelines advocate monitoring serum PSA levels every 6–12 months for the first 5 years and annually thereafter following definitive therapy. Despite these recommendations, there is currently no consensus on the optimal timing for discontinuation of PSA surveillance, particularly in patients who remain biochemically disease-free for extended periods. The Japanese Urological Association guidelines similarly emphasize PSA-based monitoring but do not provide explicit recommendations regarding the cessation of follow-up [15]. This underscores the ongoing uncertainty in defining evidence-based surveillance strategies.

HDR-BT, particularly in combination with EBRT, has consistently demonstrated favorable long-term oncologic outcomes in patients with high- and very high-risk localized PC. Randomized and large retrospective studies have shown that HDR-BT boost provides superior biochemical control compared with EBRT alone, without significantly increasing late toxicity [2,6,8,9,16,17,18,19]. For instance, Hoskin et al. demonstrated improved biochemical progression-free survival with HDR-BT boost in a randomized setting [18], and subsequent large institutional and multicenter series have reported similar benefits [2,8,9,17,20]. More recently, durable biochemical control with HDR-BT boost has been confirmed based on 24 years of follow-up studies [21]. In line with these observations, our previous study also showed excellent long-term outcomes in a cohort treated with HDR-BT + EBRT + ADT, with 7-year RFS exceeding 90% and post-neoadjuvant PSA > 0.05 ng/mL emerging as an independent predictor of adverse outcomes [22]. Collectively, these findings underscore the role of HDR-BT as a highly effective curative approach and provide a rationale for exploring PSA-based biomarkers to refine long-term follow-up strategies.

The prognostic significance of PSA nadir and long-term PSA dynamics after HDR-BT has attracted increasing attention. Several reports have shown that the depth of PSA nadir strongly correlates with long-term cancer control. Tsumura et al. demonstrated that 94% of patients achieving a PSA nadir ≤ 0.02 ng/mL after HDR-BT + EBRT + long-term ADT remained free from PSA elevation 7 years post-treatment [23]. Similarly, Nakazono et al. reported that no patients with PSA ≤ 0.2 ng/mL at 8 years experienced biochemical recurrence or clinical progression [24]. Noble et al. validated the prognostic utility of a low PSA nadir after brachytherapy in an external cohort [25], while Nagore et al. showed that a PSA nadir ≤ 0.2 ng/mL after HDR-BT monotherapy predicted excellent 10-year outcomes [26]. In addition, multiple institutional series have confirmed that both the nadir level and its durability, as well as parameters such as time to nadir and PSA fluctuations during follow-up, stratify patients according to their risk of late recurrence [27,28,29]. Collectively, these findings indicate that PSA nadir and subsequent PSA dynamics serve as powerful surrogate markers of treatment success. Consistently, long-term low PSA levels have been shown to correlate with freedom from recurrence and PC-specific mortality [30], supporting their use in guiding surveillance strategies.

In our cohort, long-term disease control after HDR-BT was excellent, and PC-specific mortality was rare. Consequently, OS was determined primarily by age and comorbidities associated with aging. This finding should not be interpreted as a lack of clinical utility of surveillance, but rather as a reflection of the competing-risk nature of the studied population. Importantly, while higher baseline PSA, advanced T stage, and higher Grade Group were significantly associated with RFS in univariate analyses, none of these parameters remained independently predictive in multivariate analysis. These findings suggest that once durable PSA control is achieved after HDR-BT, traditional baseline prognostic factors lose relevance, and long-term PSA kinetics become the principal determinant of oncologic outcome.

Our study adds to this growing body of evidence by demonstrating that PSA levels measured 5 years after HDR-BT are strongly predictive of subsequent outcomes. Importantly, patients achieving PSA ≤ 0.2 ng/mL at 7 years remained free from biochemical or clinical recurrence, irrespective of adjuvant HT. This finding indicates that achieving a stable, low PSA several years after HDR-BT may reflect a “functional cure” [30], and such patients could be candidates for de-escalated surveillance. Establishing time-bound PSA thresholds is clinically meaningful, as it provides a practical tool to identify patients at extremely low risk of recurrence. Such an approach may justify shortening the duration or intensity of follow-up in selected patients, reducing healthcare costs and alleviating the psychological burden of lifelong surveillance.

Several limitations must be acknowledged. First, the retrospective, single-center, and nonrandomized design introduces inherent selection and treatment biases. The relatively small number of oncologic events limits the statistical power to detect rare late recurrences. Second, treatment protocols were not entirely uniform during the study period, and the decision regarding the initiation and duration of ADT was at the discretion of attending physicians, which may have influenced PSA kinetics. Third, serial testosterone measurements after ADT discontinuation were not available, precluding evaluation of their effect on delayed biochemical relapse. Despite these limitations, the long follow-up period is a major strength, allowing a reliable assessment of late outcomes.

Future multicenter prospective studies with standardized treatment and follow-up protocols are warranted to validate our findings and to establish PSA-based criteria for safely discontinuing surveillance. Our long-term data indicates that patients achieving a PSA ≤ 0.2 ng/mL at 7 years after HDR-BT represent an extremely low-risk cohort for subsequent oncologic events. These findings support the possibility that, in carefully selected patients, routine PSA surveillance may be safely de-escalated or even discontinued, reducing the psychological and economic burden of lifelong follow-up without compromising oncological safety.

## 5. Conclusions

The combination of HDR-BT and EBRT provided durable long-term disease control. Our findings indicate that patients achieving a PSA ≤ 0.2 ng/mL at 7 years post-treatment represent an extremely low-risk cohort for subsequent oncologic events. In such carefully selected patients, de-escalation or even discontinuation of routine PSA monitoring may be considered, potentially reducing the burden of lifelong surveillance without compromising oncological safety.

## Figures and Tables

**Table 1:** Patient characteristics.

	All Patients	No Recurrence at 5 Years	No Recurrence at 7 Years
Number of patients	338	291	195
Follow-up (year)			
Median (range)	8.9 (5.0–19.0)	9.1 (5.0–19.0)	10.1 (7.0–19.0)
Age (year), *n* (%)			
Median (range)	69 (50–83)	69 (50–83)	67 (51–81)
<70	190 (56.2)	167 (57.4)	126 (64.6)
≥70	148 (43.8)	124 (42.6)	69 (35.4)
PSA at diagnosis (ng/mL), *n* (%)			
Median (range)	12.25 (2.26–557.64)	12.20 (2.26–557.64)	12.20 (2.59–557.64)
≤20	228 (67.5)	196 (67.4)	136 (69.7)
>20, ≤40	70 (20.7)	63 (21.6)	40 (20.5)
>40	40 (11.8)	32 (11.0)	19 (9.7)
Grade Group, *n* (%)			
≤3	153 (45.3)	131 (45.0)	97 (49.7)
4	102 (30.2)	88 (30.2)	54 (27.7)
5	82 (24.3)	71 (24.4)	43 (22.1)
Unknown	1 (0.3)	1 (0.3)	1 (0.5)
Clinical T stage, *n* (%)			
cT1	27 (8.0)	23 (7.9)	15 (7.7)
cT2	159 (47.0)	138 (47.4)	105 (53.8)
cT3	131 (38.8)	112 (38.5)	68 (34.9)
cT4	21 (6.2)	18 (6.2)	7 (3.6)
Clinical N stage, *n* (%)			
cN0	320 (94.7)	278 (95.5)	188 (96.4)
cN1	18 (5.3)	13 (4.5)	7 (3.6)
Adjuvant hormonal therapy, *n* (%)			
Yes	162 (47.9)	141 (48.5)	80 (41.0)
HDR-BT protocol, *n* (%)			
9.5Gy × 2	207 (61.2)	180 (61.9)	150 (76.9)
13Gy × 1	131 (38.8)	111 (38.1)	45 (23.1)
Pre HDR-BT PSA (ng/mL)			
Median (range)	0.057 (0–25.442)	0.053 (0–25.442)	0.046 (0–10.066)
PSA at 5 years (ng/mL)			
Median (range)	0.033 (0–3.540)	0.032 (0–1.322)	0.035 (0–0.781)

HDR-BT, high-dose-rate brachytherapy; PSA, prostate-specific antigen.

**Table 2:** Prognostic factors for recurrence after 5 years following HDR-BT.

Covariant		Recurrence-Free Survival			
		Univariate		Multivariate	
		HR (95% CI)	*p*-Value	HR (95% CI)	*p*-Value
Age (year)	<70	Reference		Reference	
	≥70	1.62 (0.41–6.50)	0.493	1.48 (0.30–7.38)	0.630
PSA at diagnosis (ng/mL)	≤20	Reference		Reference	
	>20, ≤40	2.34 (0.52–10.47)	0.266	1.54 (0.26–9.03)	0.632
	>40	1.59 (0.18–14.26)	0.678	0.14 (0.00–5.38)	0.288
Pre HDR-BT PSA (ng/mL)	≤0.05	Reference		Reference	
	>0.05	2.25 (0.45–11.17)	0.320	4.51 (0.46–44.13)	0.195
Clinical T stage	<T3	Reference		Reference	
	≥T3	4.21 (0.85–20.89)	0.079	11.66 (0.72–188.21)	0.083
Grade Group	≤3	Reference		Reference	
	4	1.66 (0.23–11.82)	0.611	6.05 (0.37–97.96)	0.205
	5	4.12 (0.75–22.54)	0.102	16.59 (0.93–295.08)	0.056
Adjuvant hormonal therapy	No	Reference		Reference	
	Yes	2.07 (0.49–8.70)	0.320	0.38 (0.04–3.77)	0.411
HDR-BT protocol	9.5Gy × 2	Reference		Reference	
	13Gy × 1	2.48 (0.61–10.11)	0.207	1.35 (0.21–8.65)	0.754
PSA at 5 years (ng/mL)	≤0.03	Reference		Reference	
	>0.03, ≤0.2	1.10 (0.15–7.80)	0.925	1.73 (0.22–13.62)	0.602
	>0.2	10.70 (1.96–58.42)	0.006	117.57 (6.22–2223.37)	0.001

CI, confidence interval; HDR-BT, high-dose-rate brachytherapy; HR, hazard ratio; PSA, prostate-specific antigen.

## Data Availability

Data is contained within the article or Supplementary Materials.

## Supplementary Materials

The following supporting information can be downloaded at: https://www.mdpi.com/article/10.3390/cancers17193151/s1, Figure S1: Recurrence-free survival after HDR-BT stratified by (a) age, (b) initial PSA, (c) clinical T stage, (d) clinical N stage, (e) Grade Group (GG), (f) pre HDR-BT PSA, and (g) HDR-BT protocol; Table S1: Prognostic factors for recurrence following HDR-BT; Table S2: The eventual recurrence stratified by PSA at 5 years after HDR-BT; Table S3: The eventual recurrence stratified by PSA at 7 years after HDR-BT.

## Abbreviations

The following abbreviations are used in this manuscript:

ADT	Androgen deprivation therapy
CSS	Cancer-specific survival
EBRT	External beam radiotherapy
HDR-BT	High-dose-rate brachytherapy
HT	Hormonal therapy
NCCN	National Comprehensive Cancer Network
OS	Overall survival
PC	Prostate cancer
PSA	Prostate-specific antigen
RFS	Recurrence-free survival
RT	Radiotherapy

## References

[1] Spratt D., Srinivas S., Schaeffer E., Adra N., Ahmed B., An Y., Bitting R., Chapin B., Cheng H., Cho S. Prostate Cancer, Version 2.2025, NCCN Clinical Practice Guidelines in Oncology. https://www.nccn.org/professionals/physician_gls/pdf/prostate.pdf.

[2] Ishiyama H., Kamitani N., Kawamura H., Kato S., Aoki M., Kariya S., Matsumura T., Kaidu M., Yoshida K., Hashimoto Y. (2017). Nationwide multi-institutional retrospective analysis of high-dose-rate brachytherapy combined with external beam radiotherapy for localized prostate cancer: An Asian Prostate HDR-BT Consortium. Brachytherapy.

[3] Yoshioka Y., Kotsuma T., Komiya A., Kariya S., Konishi K., Nonomura N., Ogawa K., Tanaka E., Nishimura K., Fujiuchi Y. (2017). Nationwide, Multicenter, Retrospective Study on High-Dose-Rate Brachytherapy as Monotherapy for Prostate Cancer. Int. J. Radiat. Oncol. Biol. Phys..

[4] Hsu I.C., Rodgers J.P., Shinohara K., Purdy J., Michalski J., Roach M., Vigneault E., Ivker R.A., Pryzant R.M., Kuettel M. (2021). Long-Term Results of NRG Oncology/RTOG 0321: A Phase II Trial of Combined High Dose Rate Brachytherapy and External Beam Radiation Therapy for Adenocarcinoma of the Prostate. Int. J. Radiat. Oncol. Biol. Phys..

[5] Makino T., Sakurai T., Takamatsu S., Iwamoto H., Yaegashi H., Iijima M., Kawaguchi S., Nohara T., Shigehara K., Izumi K. (2021). The effectiveness of high-dose-rate brachytherapy with external beam radiotherapy for clinically locally advanced and node-positive prostate cancer: Long-term results of a retrospective study. Int. J. Clin. Oncol..

[6] Slevin F., Zattoni F., Checcucci E., Cumberbatch M.G.K., Nacchia A., Cornford P., Briers E., De Meerleer G., De Santis M., Eberli D. (2023). A Systematic Review of the Efficacy and Toxicity of Brachytherapy Boost Combined with External Beam Radiotherapy for Nonmetastatic Prostate Cancer. Eur. Urol. Oncol..

[7] Astrom L., Grusell E., Sandin F., Turesson I., Holmberg L. (2018). Two decades of high dose rate brachytherapy with external beam radiotherapy for prostate cancer. Radiother. Oncol..

[8] Wedde T.B., Smastuen M.C., Brabrand S., Fossa S.D., Kaasa S., Tafjord G., Russnes K.M., Hellebust T.P., Lilleby W. (2019). Ten-year survival after High-Dose-Rate Brachytherapy combined with External Beam Radiation Therapy in high-risk prostate cancer: A comparison with the Norwegian SPCG-7 cohort. Radiother. Oncol..

[9] Hjalm-Eriksson M., Nilsson S., Brandberg Y., Johansson H., Lennernas B., Lundell G., Castellanos E., Ullen A. (2021). High rate of local control and cure at 10 years after treatment of prostate cancer with external beam radiotherapy and high-dose-rate brachytherapy: A single centre experience. Acta Oncol..

[10] Hoskin P.J., Rojas A.M., Ostler P.J., Bryant L., Lowe G.J. (2021). Randomised trial of external-beam radiotherapy alone or with high-dose-rate brachytherapy for prostate cancer: Mature 12-year results. Radiother. Oncol..

[11] Brierley J., Gospodarowicz M., Wittekind C. (2016). The TNM Classification of Malignant Tumours.

[12] Sakurai T., Takamatsu S., Shibata S., Iwata K., Taka M., Gabata T., Kumano T., Makino T., Mizokami A. (2020). Toxicity and clinical outcomes of single-fraction high-dose-rate brachytherapy combined with external beam radiotherapy for high-/very high-risk prostate cancer: A dosimetric analysis of toxicity. Jpn. J. Radiol..

[13] Roach M., Hanks G., Thames H., Schellhammer P., Shipley W.U., Sokol G.H., Sandler H. (2006). Defining biochemical failure following radiotherapy with or without hormonal therapy in men with clinically localized prostate cancer: Recommendations of the RTOG-ASTRO Phoenix Consensus Conference. Int. J. Radiat. Oncol. Biol. Phys..

[14] National Cancer Center Japan. Cancer Information Service. Prostate Cancer. https://ganjoho.jp/public/cancer/prostate/index.html.

[15] Kohjimoto Y., Uemura H., Yoshida M., Hinotsu S., Takahashi S., Takeuchi T., Suzuki K., Shinmoto H., Tamada T., Inoue T. (2024). Japanese clinical practice guidelines for prostate cancer 2023. Int. J. Urol..

[16] Slevin F., Rodda S.L., Bownes P., Murray L., Bottomley D., Wilkinson C., Adiotomre E., Al-Qaisieh B., Dugdale E., Hulson O. (2020). A comparison of outcomes for patients with intermediate and high risk prostate cancer treated with low dose rate and high dose rate brachytherapy in combination with external beam radiotherapy. Clin. Transl. Radiat. Oncol..

[17] Demanes D.J., Rodriguez R.R., Schour L., Brandt D., Altieri G. (2005). High-dose-rate intensity-modulated brachytherapy with external beam radiotherapy for prostate cancer: California endocurietherapy’s 10-year results. Int. J. Radiat. Oncol. Biol. Phys..

[18] Hoskin P.J., Rojas A.M., Bownes P.J., Lowe G.J., Ostler P.J., Bryant L. (2012). Randomised trial of external beam radiotherapy alone or combined with high-dose-rate brachytherapy boost for localised prostate cancer. Radiother. Oncol..

[19] Khor R., Duchesne G., Tai K.H., Foroudi F., Chander S., Van Dyk S., Garth M., Williams S. (2013). Direct 2-arm comparison shows benefit of high-dose-rate brachytherapy boost vs external beam radiation therapy alone for prostate cancer. Int. J. Radiat. Oncol. Biol. Phys..

[20] Miszczyk M., Magrowski L., Krzysztofiak T., Stando R., Majewski W., Stawiski K., Masri O., Ciepal J., Depowska G., Chimiak K. (2023). Brachytherapy boost improves survival and decreases risk of developing distant metastases compared to external beam radiotherapy alone in intermediate and high risk group prostate cancer patients. Radiother. Oncol..

[21] Prada Gomez P.J., Rivero Perez A.L., Carballido Rodriguez J., Anchuelo Latorre J., Fabregat Borras R., Gutierrez Ruiz M., Rodriguez-Acosta Caballero C., Carrascal Gordillo C.F., Galdos Barroso M.P., Navarrete Solano P.A. (2025). Long-Term Outcomes After High-Dose-Rate Brachytherapy and Hypofractionated External Beam Radiotherapy in Very High-Risk Prostate Cancer: A 24-Year Follow-Up. Biomedicines.

[22] Makino T., Sakurai T., Takamatsu S., Kano H., Naito R., Iwamoto H., Yaegashi H., Kawaguchi S., Shigehara K., Nohara T. (2025). Biochemical response to neoadjuvant hormonal therapy predicts long-term prostate cancer survival outcomes after high-dose-rate brachytherapy with external beam radiotherapy. Sci. Rep..

[23] Tsumura H., Satoh T., Ishiyama H., Tabata K., Komori S., Sekiguchi A., Ikeda M., Kurosaka S., Fujita T., Kitano M. (2016). Prostate-specific antigen nadir after high-dose-rate brachytherapy predicts long-term survival outcomes in high-risk prostate cancer. J. Contemp. Brachytherapy.

[24] Nakazono M., Urabe F., Iwatani K., Imai Y., Tashiro K., Honda M., Koike Y., Shimomura T., Sato S., Takahashi H. (2023). Patients with PSA below 0.2 ng/mL at 8 years post high-dose-rate brachytherapy have an extremely low risk of subsequent recurrence. Int. J. Urol..

[25] Noble D.J., Doyle E., Tramonti G., Law A.B., Sundaramurthy A., Brush J.P., Keanie J., Wood C., Drewell P., Keough W. (2022). Defining Biochemical Cure After Low Dose Rate Prostate Brachytherapy: External Validation of 4-year Prostate-specific Antigen Nadir as a Predictor of 10- and 15-year Disease-free Survival. Clin. Oncol..

[26] Nagore G., Moreno-Olmedo E., Suarez-Gironzini V., Aakki L., Li R.G., Gomez E., Garcia A., Beltran L., Gomez-Iturriaga A. (2023). Long-term outcomes of ultra-hypofractionated 2 fractions single day HDR brachytherapy in localized prostate cancer. Radiother. Oncol..

[27] Geara F.B., Bulbul M., Khauli R.B., Andraos T.Y., Abboud M., Al Mousa A., Sarhan N., Salem A., Ghatasheh H., Alnsour A. (2017). Nadir PSA is a strong predictor of treatment outcome in intermediate and high risk localized prostate cancer patients treated by definitive external beam radiotherapy and androgen deprivation. Radiat. Oncol..

[28] Coelho M.O., Dal Col L.S., Capibaribe D.M., Salgado C.M., Travassos T.C., Junior V.J., Monti C.R., Reis L.O. (2022). PSA nadir predicts biochemical recurrence after external beam radiation therapy combined to high dose rate brachytherapy in the treatment of prostate cancer. Am. J. Clin. Exp. Urol..

[29] Miszczyk M., Magrowski L., Masri O., Jablon’ska I., Nowicka Z., Krzysztofiak T., Wojcieszek P., Lipka-Rajwa A., Ciepal J., Depowska G. (2022). Prostate-specific antigen kinetics and metastasis-free survival in patients treated with external beam radiotherapy combined with high-dose-rate brachytherapy boost and androgen deprivation therapy for localized prostate cancer. J. Contemp. Brachytherapy.

[30] Crook J.M., Tang C., Thames H., Blanchard P., Sanders J., Ciezki J., Keyes M., Morris W.J., Merrick G., Catton C. (2020). A biochemical definition of cure after brachytherapy for prostate cancer. Radiother. Oncol..

