# Supplementary material for: Seven-Year PSA ≤ 0.2 ng/mL After High-Dose-Rate Brachytherapy Indicates Eligibility for Discontinuing PSA Surveillance in Prostate Cancer
Source: Cancers (Basel). 2025 Sep 28;17(19):3151. doi: 10.3390/cancers17193151 (PMC12523830; doi:10.3390/cancers17193151)
Supplement: Supplementary file 1 [file cancers-17-03151-s001.zip › Supplementary Figure.pptx]

## Slide 1
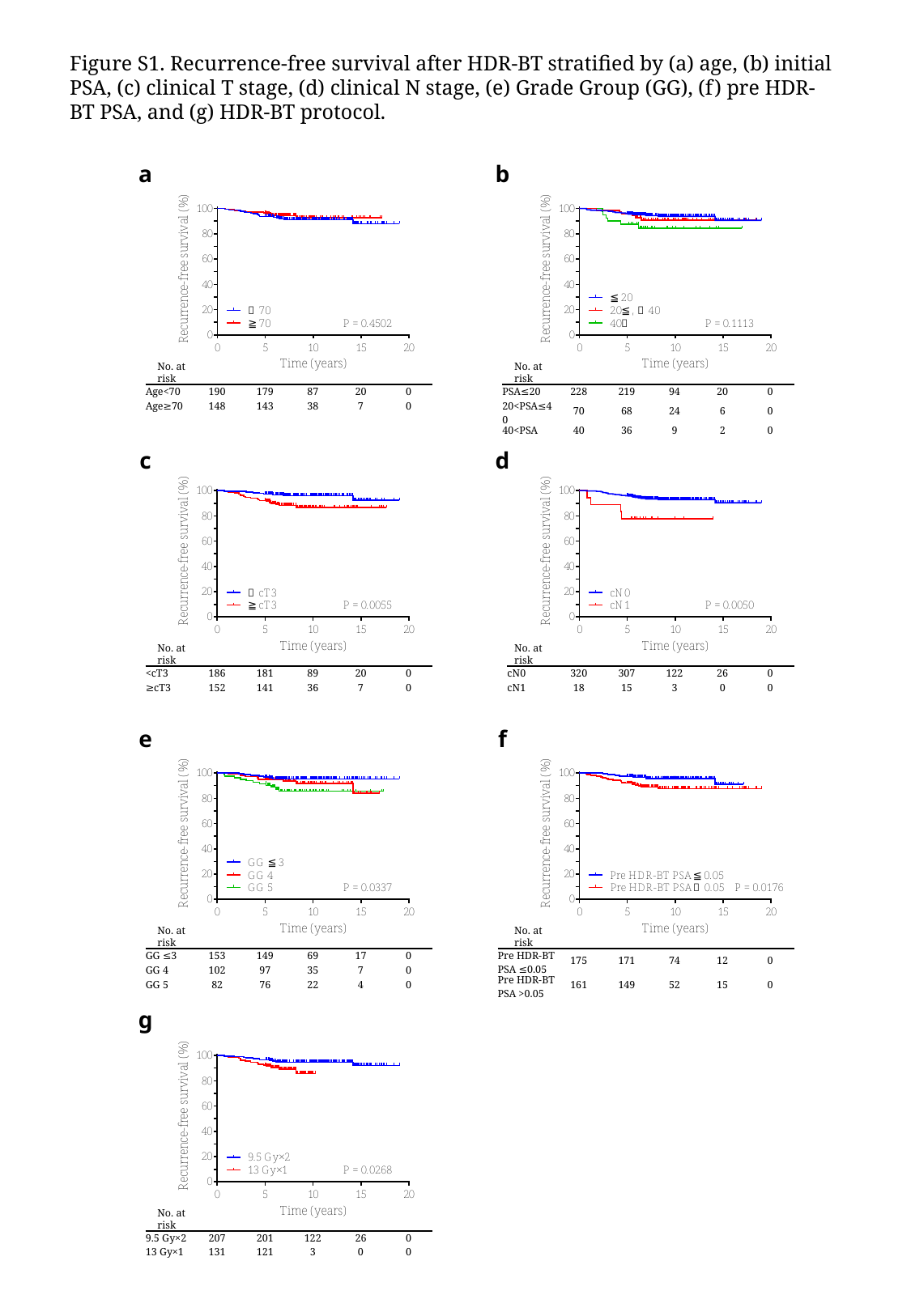

Figure S1. Recurrence-free survival after HDR-BT stratified by (a) age, (b) initial PSA, (c) clinical T stage, (d) clinical N stage, (e) Grade Group (GG), (f) pre HDR-BT PSA, and (g) HDR-BT protocol.
a
b
No. at risk
No. at risk
| Age<70 | 190 | 179 | 87 | 20 | 0 |
| --- | --- | --- | --- | --- | --- |
| Age≥70 | 148 | 143 | 38 | 7 | 0 |
| PSA≤20 | 228 | 219 | 94 | 20 | 0 |
| --- | --- | --- | --- | --- | --- |
| 20<PSA≤40 | 70 | 68 | 24 | 6 | 0 |
| 40<PSA | 40 | 36 | 9 | 2 | 0 |
c
d
No. at risk
No. at risk
| <cT3 | 186 | 181 | 89 | 20 | 0 |
| --- | --- | --- | --- | --- | --- |
| ≥cT3 | 152 | 141 | 36 | 7 | 0 |
| cN0 | 320 | 307 | 122 | 26 | 0 |
| --- | --- | --- | --- | --- | --- |
| cN1 | 18 | 15 | 3 | 0 | 0 |
e
f
No. at risk
No. at risk
| GG ≤3 | 153 | 149 | 69 | 17 | 0 |
| --- | --- | --- | --- | --- | --- |
| GG 4 | 102 | 97 | 35 | 7 | 0 |
| GG 5 | 82 | 76 | 22 | 4 | 0 |
| Pre HDR-BT PSA ≤0.05 | 175 | 171 | 74 | 12 | 0 |
| --- | --- | --- | --- | --- | --- |
| Pre HDR-BT PSA >0.05 | 161 | 149 | 52 | 15 | 0 |
g
No. at risk
| 9.5 Gy×2 | 207 | 201 | 122 | 26 | 0 |
| --- | --- | --- | --- | --- | --- |
| 13 Gy×1 | 131 | 121 | 3 | 0 | 0 |
